# Sound transmission in human thorax through airway insonification: an experimental and computational study with diagnostic applications

**DOI:** 10.1007/s11517-020-02211-y

**Published:** 2020-07-14

**Authors:** Harish Palnitkar, Brian M. Henry, Zoujun Dai, Ying Peng, Hansen A. Mansy, Richard H. Sandler, Robert A Balk, Thomas J. Royston

**Affiliations:** a) Department of Mechanical and Industrial Engineering, University of Illinois at Chicago, Chicago, IL 60607, USA;; b) Richard and Loan Hill Department of Bioengineering, University of Illinois at Chicago, Chicago, IL 60607, USA;; c) University of Central Florida, Orlando, FL 32816, USA;; d) Rush University Medical Center, Chicago, IL 60612, USA

**Keywords:** computational modeling, finite element analysis, lung acoustics, pneumothorax, fibrosis, tumor

## Abstract

Pulmonary diseases and injury lead to structural and functional changes in the lung parenchyma and airways, often resulting in measurable sound transmission changes on the chest wall surface. Additionally, noninvasive imaging of externally driven mechanical wave motion in the chest (e.g., using magnetic resonance elastography) can provide information about lung stiffness and other structural property changes which may be of diagnostic value. In the present study, a comprehensive computational simulation (in silico) model was developed to simulate sound wave propagation in the airways, parenchyma, and chest wall under normal and pathological conditions that create distributed structural (e.g., pneumothoraces) and diffuse material (e.g., fibrosis) changes, as well as a localized structural and material changes as may be seen with a neoplasm. Experiments were carried out in normal subjects to validate the baseline model. Sound waves with frequency content from 50 to 600 Hz were introduced into the airways of three healthy human subjects through the mouth, and transthoracic transmitted waves were measured by scanning laser Doppler vibrometry at the chest wall surface. The computational model predictions of a frequency-dependent decreased sound transmission due to pneumothorax were consistent with experimental measurements reported in previous work. Predictions for the case of fibrosis show that while shear wave motion is altered, changes to compression wave propagation are negligible, and thus insonification, which primarily drives compression waves, is not ideal to detect the presence of fibrosis. Results from the numerical simulation of a tumor show an increase in the wavelength of propagating waves in the immediate vicinity of the tumor region.

## Graphical Abstract

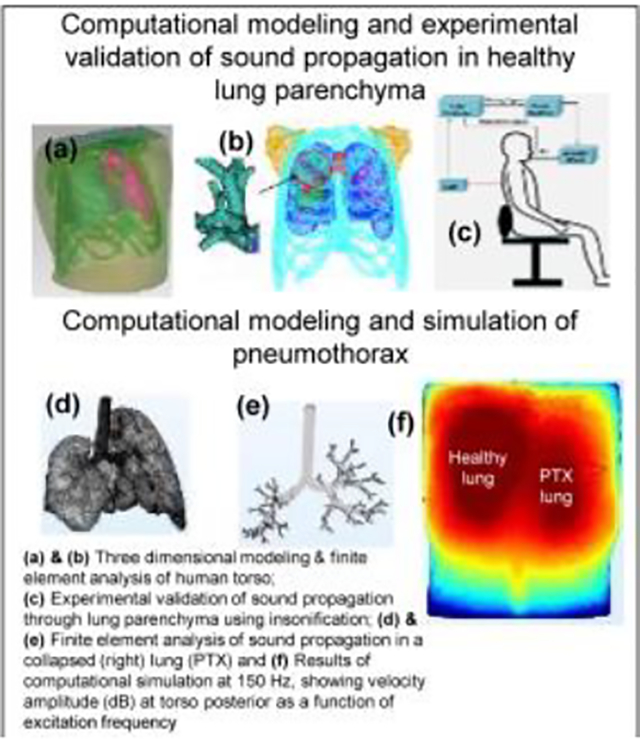

## 1. Introduction

### 1.1 Motivation

The lungs are comprised of soft tissue, airways, alveoli and vasculature structures, acoustically behaving as a poroviscoelastic material over a broad frequency range [1]. Due to this fine-structure heterogeneity, conventional imaging modalities such as magnetic resonance imaging (MRI), ultrasound (US) and X-ray computed tomography (CT) have limited resolution to detect and quantify changes in the anatomy and properties in vivo that may be indicative of many pulmonary pathologies, including subtle fibrosis, inflammation, tumors, pneumonia and pneumothorax [2]. For instance, US is limited to detecting anomalies on the periphery of the lung due to the acoustic impedance mismatch between the lung parenchyma and chest wall. CT has the disadvantage of ionizing radiation risk, while both CT and MRI are costly and provide limited soft tissue contrast for subtle or small changes.

Nonetheless, a phase-contrast based MRI technique, known as Magnetic Resonance Elastography (MRE), which involves simultaneous application of harmonic excitation to the organ/tissue under consideration in the presence of a radio frequency (RF) pulse (at the same frequency as that of the mechanical excitation), has demonstrated utility in the assessment of pulmonary pathology [3–5]. That said, MRE of the lungs is challenging since the pulmonary system is a complex poroviscoelastic structure with a significant amount of air, unlike other biological structures such as heart [6], brain [7] and muscles [8]. A better understanding of propagation of mechanical waves inside the pulmonary structures may help improve the utility of MRE for diagnosis and monitoring of disease states. This understanding can be achieved through the development of an experimentally validated comprehensive computational model of sound propagation inside the human torso, as described in this article.

Another non-invasive technique, known as the forced oscillation technique (FOT) [9], involves application of forced pressure oscillations and measurement of air flow into/out of the lungs. The pressure input is introduced either at the opening of airways or at the chest wall. The FOT focuses on acquiring an impedance measurement, by measuring the ratio of the forced input to the resulting air flow. While similar, the techniques of insonification and percussion (defined and explained in section 1.2) used in the present study, focus not on impedance but rather on how sound is transmitted throughout the torso region, and ultimately measured on the torso surface.

## 1.2 Prior work

Prior research from this group has involved computational modeling and simulation, coupled with experimental validation of sound propagation in human and porcine subjects using the excitation techniques of insonification on porcine subjects [10] and percussion applied at the sternum on porcine and human subjects [11]. Insonification refers to introducing sound into the airways via an external acoustic source. This sound in turn leads to propagation of mechanical waves throughout the torso of the subject. Percussion involves introduction of mechanical wave motion on the torso surface, such as at the sternum. For both of these techniques, the resulting wave motion can be measured at the torso posterior surface by using a digital stethoscope or a scanning laser Doppler vibrometer (SLDV) [12].

Dai et al [13] performed a range of experiments on porcine lungs to determine key attributes of mechanical wave propagation in lungs, namely, compression and surface wave speed and attenuation, and used them to compare against the values predicted by an adaptation of Biot theory and an “effective medium” theory. It was concluded that the predictions made by Biot theory were closer to the experimental measurements. The same authors [14] developed an analytical technique to compute airway acoustic transmission in bifurcating airway segments and validated the technique through experimentation on a viscoelastic phantom containing a network of branching airways. Henry et al [15] extended this analytical approach to more complex and realistic airway models with an emphasis on investigating the influence of respiratory pathologies such as pulmonary fibrosis, bronchoconstriction and pulmonary infiltrate on mechanical and geometrical properties of the complex airway tree models. Peng et al applied these modeling and analytical techniques and experimentally and computationally investigated sound transmission in the porcine thorax using airway “insonification” [10] and percussion applied at the sternum [11]. Mathematical models of sound transmission inside the pulmonary system have been also developed by subdividing the system into airways and lung parenchyma [16]. The current study, building upon this prior work, investigates the effect of different conditions – fibrosis, pneumothorax and tumor – on sound transmission in the human thorax using airway “insonification”.

### 1.2.1 Modeling of the airways

A direct method of developing realistic geometrical models of the larger airways is through the use of CT images [17]. However, small conducting and terminal airways cannot be imaged with clarity. As a result, there are alternative methods of generating airway geometries based on mathematical algorithms. One of the first mathematical models for characterizing airway segments and branching behavior was developed by Horsfield *et al* [18]. Kitaoka *et al* [19] proposed a deterministic algorithm that could generate an elaborate and complex 3D airway geometry with the number of segments specified by the user in a non-symmetric manner. The current study uses an airway mesh that was developed and shared by Dr. Kitaoka’s research group: this model was based on the seven fundamental laws of branching. The branching angle was restricted to 90 degrees. This model was not subject specific, but rather, a computational one. The authors recommend the usage of a newer and more accurate subject specific model of airways, developed by Henry et al [20], that uses CT images of the human subject in conjunction with computer algorithm.

### 1.2.2 Modeling of the lung parenchyma

In order to predict the mechanical wave motion inside the lung parenchyma for wavelengths that are greater than the dimensions of the microscopic heterogeneous features of the lung, homogenized representations of the lung’s mechanical properties have been used [21]. Based on these homogenized representations, two models of wave propagation through the lung parenchyma have been proposed: the first model is based on the “bubble swarm” theory (also known as “effective medium” theory) [22] while the second model uses the Biot theory of poroelasticity [16]. The latter approach that uses Biot theory results in a better prediction of the behavior of mechanical waves as compared to the effective medium approach. The current work models the material properties of the lung parenchyma using the Biot theory.

## 1.3 Objective

The goal of this study is two-fold: firstly, to perform in-vivo experimentation on 3 healthy human subjects using the acoustical excitation technique of airway “insonification” (section 4.2.1.3) and perform measurements of the resulting surface velocity on the posterior side of the torso using a scanning laser Doppler vibrometer (SLDV). The second and more significant aim of this work is to develop and experimentally validate a computational model of sound transmission inside human lungs that utilizes parenchymal and major airway geometry developed from CT scans as described in section 4.1. This computational model is essentially subject-based, and thus captures the variation in the geometric parameters of the pulmonary system from one person to another. A quantitative comparison of the sound propagation inside the lung parenchyma using in vivo experimentation and computational simulation via finite element analysis (FEA) is performed in order to evaluate the computational model. Finally, this subject-based computational model is used to perform finite element analysis in order to simulate specific pathological cases in the lung parenchyma associated with pneumothorax, diffuse fibrosis and a localized tumor. FEA is used as a tool to develop unique “cross-sectional” views of the torso in order to visualize the nature of propagation of mechanical waves inside healthy as well as diseased lung parenchyma.

### 1.3.1 Rationale for the present choice of pulmonary pathologies

The current work considers three pathologies that are expected to have substantially different effects on sound transmission. Pneumothorax involves an alteration in lung parenchymal geometry and density, as well as the introduction of trapped air between the parenchyma and chest wall. This should have a substantial effect on transmission. Fibrosis results in a distributed change in shear viscoelastic properties in the parenchyma with no significant change in geometry. A tumor results in a localized change in both tissue shear viscoelastic properties and density.

Pneumothorax is defined as an abnormal accumulation of air in the region between the lungs and the pleural space that results in a partial or complete collapse of the lung. Pneumothorax may be caused by penetrating or blunt chest trauma (e.g. knife wounds or car accidents) or may result from small sacs of air (blebs) in lung tissue that rupture, causing air to leak into the pleural space. The trapped air in the pleural space can increase the intra- pleural pressure and lead to lung collapse. Pneumothorax is more common in men than in women. This condition occurs in 7.4 to 18 per 100,000 men and 1.2 to 6 per 100,000 women each year [23].

Idiopathic pulmonary fibrosis is a chronic, progressive lung disease where lung parenchyma becomes fibrotic, thereby compromising oxygenation of the blood. This scarring typically worsens over time until inadequate oxygenation leads to death. Most individuals survive 3 to 5 years after their diagnosis. In the United States, about 100,000 people are affected with 30,000 to 40,000 new cases diagnosed yearly [24].

Poor diagnostic sensitivity and specificity underlie the reason that lung cancer remains the leading cause of cancer death in both the United States and in the world for both men and women. Nearly 80% of lung cancer is detected at an advanced inoperable stage, while current systemic therapy offers only modest benefit. Regardless of the lung cancer type – small-cell or non-small-cell carcinoma squamous or adenocarcinoma – the tumorous growths alter the acoustic field in the lungs, as a result of changes in tissue density and viscoelasticity as compared to surrounding healthy lung tissue [25].

### 1.4 Relevance of the current study

Computational modelling has been used to better understand a wide variety of pulmonary function tests for several years. In the past year or two, researchers have been able to demonstrate commendable success in validation and usage of computational models in order to gain an insight into pulmonary diseases from a clinical point of view. For example, in 2019, the research group led by Tawhai and Lin (Choi et al [26]) developed a one dimensional CFD model to simulate the flow and pressure distribution of air in a healthy and an asthmatic lung and validated their results experimentally on 5 healthy and 5 asthmatic human subjects. The study determined flow distribution patterns inside healthy and asthmatic lungs and these experimentally validated flow distribution patterns can be used for imposing boundary conditions of three-dimensional CFD.

In the current work, the computational model aims to be more precise in comparison to the standard pulmonary function tests (PFT) by incorporating more region specific information with respect to the change of tissue viscoelastic properties due to pathologies such as PTX, fibrosis and tumors. The current work complements earlier research led by Tawhai et al [26] and lays down the scope and foundation for a more region-specific and precise assessment of mechanical wave motion inside human lung parenchyma by incorporating a detailed airway structure.

## 2. Theory

### 2.1 Airway acoustics: Impedance

Acoustic impedance is defined as the ratio of acoustic pressure to acoustic particle velocity and is a function of frequency. The acoustical impedance of airways was calculated by Habib *et al* [27, 28] using the modified Horsfield model. This model takes into account terminal respiratory tissues and assumes airway walls to be non-rigid. This section details the mathematical procedure used to determine the acoustical impedance of the airways.

#### 2.1.1 Calculation of acoustical impedance using modified Horsfield model

As described in section 1.2.1, a deterministic algorithm [29] was used to generate airways using Matlab followed by the creation of a finite element model using Ansys ICEM CFD. This section describes the mathematical technique used to compute the terminal impedance of each of the airway segments using the modified Horsfield model. The modified Horsfield model uses a “bottom-up” approach in calculating the acoustical impedance of the airway tree. For instance, the model first calculates the input acoustical impedance of a terminal (end) bronchiole (*n* = order number = 1). The model then “moves up” to the next higher level of bronchiole (i.e., larger airway) to iteratively calculate the acoustical impedance for the remainder of the airway tree, ending at *n* = 35 for the trachea. The degree of asymmetry at each airway bifurcation is specified by a recursion index denoted by Δ^(*n*)^. Therefore, an airway of order *n* bifurcates into two airways of order *n* – 1 and *n* – 1 – Δ^(*n*)^. For a particular airway having an order *n*, the bifurcated airway types (the daughter airways) are the same throughout the entire lung [16].

In general, for an *n*^th^ airway segment, the input acoustical impedance $Z_{in}^{\left(n\right)}[\omega ]$ at the end located towards the trachea is given by the expression (with *n* = 1, 2, …, 35):

…(2.1) $$Z_{in}^{\left(n\right)}[\omega ]=\frac{Z_{T}^{n}[\omega ]+Z_{0}^{n}[\omega ]\text{tanh}\left[\gamma_{0}^{\left(n\right)}[\omega ]l^{\left(n\right)}\right]}{1+\left(Z_{T}^{n}[\omega ]/Z_{0}^{n}[\omega ]\right)\text{tanh}\left[\gamma_{0}^{\left(n\right)}[\omega ]l^{\left(n\right)}\right]}$$

In the eq. (2.1), *ω* is the mechanical frequency in radians/second, $Z_{0}^{\left(n\right)}[\omega ]$ is the characteristic impedance of the *n*^th^ airway segment and $\gamma_{0}^{\left(n\right)}[\omega ]$ is the propagation coefficient of the same airway segment [30]. In addition, $Z_{T}^{\left(n\right)}[\omega ]$ is the output acoustical impedance of an *n*^th^ airway segment, at the end away from the trachea. This is given by:

…(2.2) $$Z_{T}^{\left(n\right)}[\omega ]=\{\begin{array}{cc} \frac{N_{T}}{j\omega C_{g}+1/\left[R_{t}+j\left(\omega I_{t}-1/\left[\omega C_{t}\right]\right)\right]} & n=1\\ \frac{1}{1/Z_{in}^{\left(n-1\right)}[\omega ]+1/Z_{in}^{\left(n-1-\Delta [n]\right)}[\omega ]} & n=2,\ldots ,35 \end{array}$$

In eq. (2.2) above, *N*_*T*_ denotes the number of end segments (segments with order number = *n* = 1). For any intermediate airway segment,

…(2.3) $$N_{T}^{\left(n\right)}=N_{T}^{\left(n-1\right)}+N_{T}^{\left(n-1-\Delta [n]\right)}$$

And, $Z_{T}^{\left(1\right)}=1$ and $Z_{T}^{\left(n\right)}=N_{T}$. In addition, in eq. (2.2), *R*_*t*_ represents the resistance, *I*_*t*_ the inertia and *C*_*t*_ the compliance of terminal tissue; *C*_*g*_ represents the alveolar gas compression compliance (Dubois six-element terminal airway model [31]). For the current model, *N*_*T*_ evaluates to 2.35 × 10^6^

### 2.2 Parenchymal acoustics

The lung parenchyma is a poroviscoelastic medium. There are two theories that model the transmission of sound inside the lung parenchyma, namely, the Biot Theory and the “effective medium” or “bubble swarm” theory. It has been demonstrated through experimentation [16] that the Biot theory is more accurate in predicting compression wave speed and attenuation. The dynamic oscillatory displacement *u* of the lung parenchyma is determined by solving the second-order differential equation [32]:

…(2.4) $$\mu u_{i,jj}+\left(K_{b}+\frac{\mu }{3}\right)\mu_{j,ij}-(\alpha -\beta )p_{,i}=\frac{d^{2}}{dt^{2}}\left(\rho -\beta \rho_{f}\right)u_{i}$$

Similarly, the dynamic pressure (*p*) of the air inside the lung is given by the solution to the second order differential equation [32]:

…(2.5) $$\beta p_{,ii}-\frac{\phi^{2}}{R}\rho_{f}\frac{d^{2}p}{dt^{2}}=\rho_{f}\frac{d^{2}}{dt^{2}}(\alpha -\beta )u_{i,i}$$

In equations (2.4) and (2.5), *μ* and *K*_*b*_ are the shear modulus and the bulk modulus of the lung parenchyma respectively; *ρ*_*f*_ and *ρ*are the densities of the air and the lung parenchyma respectively; *ϕ* is the volume fraction of the air inside the lung; *α*, *β* and *R* are parameters that couple the lung parenchyma with the air inside them. Based on equations (2.4) and (2.5):

…(2.6) $$c_{s}=\sqrt{\frac{\mu }{\rho -\beta \rho_{f}}}\approx \sqrt{\frac{\mu }{\rho }}$$

Note, *c*_*s*_ is the frequency-dependent complex-valued shear wave speed. This is due to the fact that the lung parenchyma is viscoelastic, thereby leading to a complex shear modulus. Thus, in equation (2.6) above, the shear modulus *μ* is a function of the frequency:

…(2.7) $$c_{s}(\omega )\approx \sqrt{\frac{\mu (\omega )}{\rho }}=\sqrt{\frac{\mu_{R}+i\mu_{I}}{\rho }}$$

Equations (2.4) and (2.5) are coupled and this leads to two compression waves [16]: a fast compression wave and a slow compression wave having a much larger attenuation. For the fast compression wave with wavenumber *k*_*pf*_, the wave propagation speed is given by:

…(2.8) $$c_{pf}=\frac{\omega }{\text{real}\left[k_{pf}\right]}$$

On the other hand, for the slow compression wave with wavenumber *k*_*ps*_, the propagation speed is given by:

…(2.9) $$c_{ps}=\frac{\omega }{\text{real}\left[k_{ps}\right]}$$

Both of these speeds are frequency dependent. In the present study, due to the relative motion between the lung parenchyma and the air inside of it, the slow compression wave attenuates faster than the fast compression wave. Only the fast compression waves propagate in the lung parenchyma [16].

## 3. In vivo experiments on healthy human subjects

**Figure 1.**
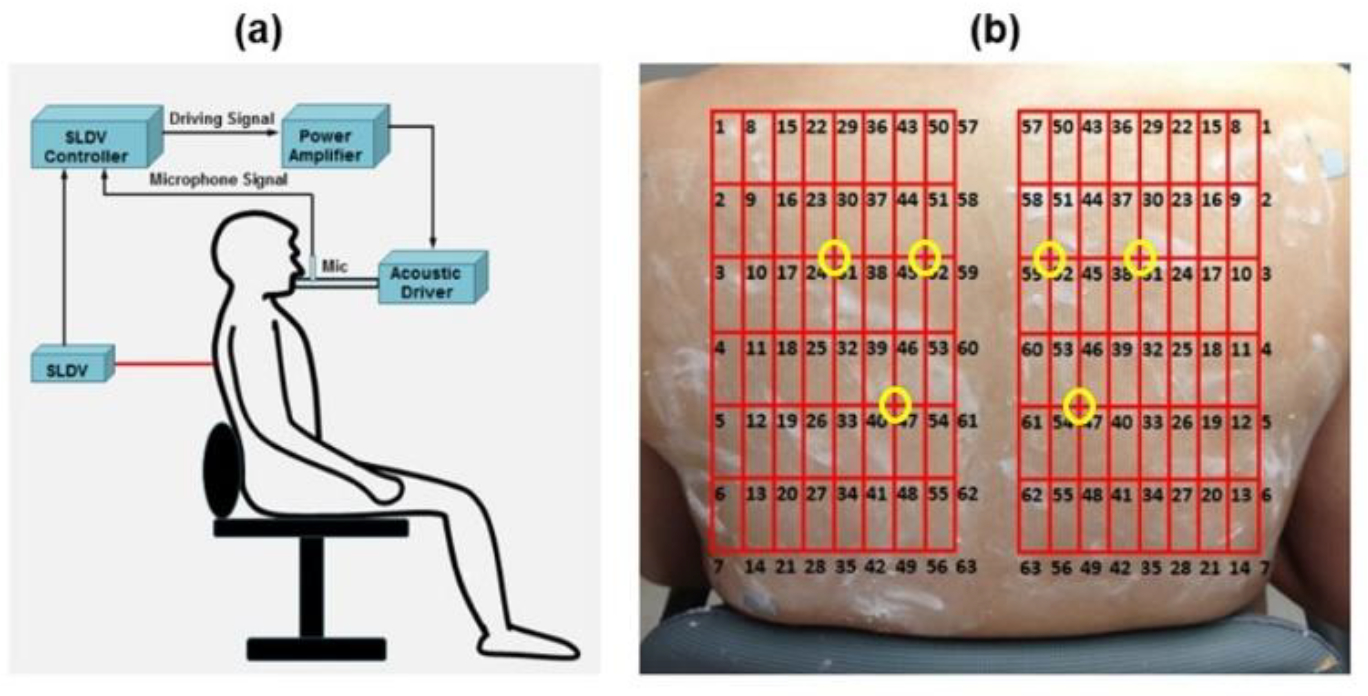
(a) Schematic diagram of the experimental setup; (b) The grid of measurement points on the dorsal side of torso of the human subject

Experimental studies were performed on three healthy male human subjects, designated as HS1, HS2 and HS3, aged 25, 28 and 26 years; and with a BMI of 21.2, 21.5 and 21.4, respectively, after receiving appropriate Institutional Review Board (IRB) approval. Figure 1 (a) shows a schematic representation of the experimental setup with the healthy human subject seated in an upright position on a chair. In order to improve the signal to noise ratio (SNR), the human subject (HS) was requested to sit still and hold breath for 20 seconds during the experiment. Retro-reflective glass beads with a nominal diameter of 45 – 63 *μm* (P-RETRO-250, Polytec, Irvine, CA) were applied externally on the thorax that was intended for measurements (the red grid shown in fig. 1 (b)) in order to increase the reflection of the laser beam emanating from a Scanning Laser Doppler Vibrometer (SLDV) (PSV-400, Polytec, Irvine, CA). A polyurethane tubing with an outer diameter of 29/32” and an inner diameter of 5/8” (McMaster-Carr, Elmhurst, IL, USA) was kept in the mouth of the human subject and inserted as deep as possible (while at the same time ensuring the safety and comfort of the human subject). Acoustic energy generated from a 3.5-inch speaker (PDWR30W, PylePro, Brooklyn, NY) was guided through this tube into the mouth and transmitted into the lungs via the airways, finally propagating into the torso. A ¼-inch microphone (377A01, PCB Piezotronic, Depew, NY) capable of measuring sound pressure level was mounted at the outlet of the tube at the mouth of human subject; the signal recorded by this microphone was used as a reference for the SLDV. The speaker was driven by a waveform generator (SignalCalc ACE, Data Physics, San Jose, CA) that generated a broad-band periodic chirp signal with a spectral content from 50 – 600 Hz. The output of the waveform generator was connected to a power amplifier (P 3500S, Yamaha, Buena Park, CA). 63 scanning points (as shown in fig. 1b) were chosen on each side of the dorsal side of the torso of the human subject.

The SLDV system acquired the surface velocity (mm/s) at each point on the measurement grid shown in fig. 1 (b) and used this data to determine the frequency response function (FRF) at each point of measurement. The FRF is defined as the frequency-dependent ratio (in absolute scale) of the output velocity measured by the SLDV over the reference pressure measured by the microphone placed close to the mouth of the human subject.

…(3.1) $$FRF=20{\text{log}}_{10}\left(\frac{\text{surface velocity measured by }\;\text{SLDV}}{P_{\text{ref}}}\right)$$

It is to be noted that the SLDV system measured the surface velocity at each of the point on the grid individually, beginning from the left-hand side (point no. 1 on the left grid) and ending on the point no. 63 on the left grid, before moving on to the grid on the right side (point no. 1) and continuing the measurements until point no. 63. This method of measuring the velocity first on the left grid, followed by the right grid, may have caused a certain amount of error (in the form of noise) during the measurement of velocity at the points on the right grid due to the inability of the human subject to hold the breath and stay still for a longer period of time, as is evident from the results discussed in section 5.1 (fig. 6).

## 4. Computational study using Finite Element Analysis

### 4.1 Three dimensional modeling

A comprehensive three dimensional model of the human torso was generated using the CT images of a male human subject obtained online from the Visible Human Project database repository [33] of the National Library of Medicine (NLM). In order to construct the three dimensional geometries from the CT images, the CT image sets were imported and processed using Mimics v.14 (Materialise, Plymouth, MI), a commercial image processing and 3D modeling software. The generated 3-D model contained the geometry of the torso, the lungs, the ribcage, the cartilage, and the scapulae. The dimensions of the human model were rescaled to appreciate the average size of the human subjects in the experimental study.

The three dimensional models thus generated were then imported into ANSYS ICEM CFD 12.1 (Ansys Inc, Canonsburg, PA), a finite element mesh generation tool [34] which was used to generate a finite element model. This finite element model was then combined with the airway mesh generated using the deterministic algorithm developed by Kitaoka et al [29]. The surface of the trachea and the mainstem bronchi were extruded in the radial direction in order to create thickness of the airways. All the further generations of airways downstream of the mainstem bronchi were assumed to have a negligible thickness. This finite element model was then imported into Comsol (Comsol Group, Stockholm, Sweden), and material properties were assigned to each organ (component) as described in the section 4.2. The finite element mesh consisted of 689 839 tetrahedral elements (3D), 144 551 triangular elements (2D), 27 713 edge elements (1D) and 9 112 vertex elements. The minimum element quality was 0.07269 and the average element quality was 0.6168. The element volume ratio was 3.781 × 10^−7^ and the mesh volume was 3.426 × 10^7^ mm^3^. These parameters were optimized with regards to the accuracy of the solution vis-à-vis the computational time, based on our earlier experiences on modeling pulmonary acoustics in porcine and human computational analysis and studies [13, 14, 18]. The three dimensional computational model of the healthy lung had 36 608 triangular surface elements and a mesh area of a 95 370 mm^3^; the lung affected with PTX had 16 476 triangular surface elements and a mesh area of 42 600 mm^3^.

### 4.2 Material properties of the Bone and soft tissue

This section explains the calculation of material properties that were assigned to bones (this includes the cartilage as well as the scapulae) and the soft tissue of the torso. The material properties of the normal lung, as well as the lung affected with pneumothorax (section 4.2.1.2), fibrosis (section 4.3.3.2) and a lung containing local tumor (section 4.2.2.2) have been explained separately.

The chest wall was considered to be a viscoelastic medium and a Voigt model of viscoelasticity was used to determine the frequency dependent shear modulus of it [35, 36] (as shown in equation (4.1) below:

…(4.1) $$\mu_{t}=\mu_{t1}+j\omega \mu_{t_{2}}$$

In eq. (4.1), the subscript “t” denotes the soft tissue; *μ*_*t*1_ and *μ*_*t*2_ denote the shear elasticity and shear viscosity with the values being *μ*_*t*1_ = 2.5 × 10^3^Pa and *μ*_*t*2_ =15 Pa.s. Assuming a density of 1100 kg/m^3^, the complex shear wave speed can then be estimated through equation (2.7) and the compression wave speed can be estimated using equation (2.9). The complex Young’s modulus of the bone can be represented by

…(4.2) $$E=E_{b1}+j\cdot E_{b2}$$

with tan *δ*_*E*_ =*E*_*b*2_ / *E*_*b*1_ [37] and subscript “b” denotes the bone.

The value of the factor tan *δ*_*E*_ is frequency-dependent and was experimentally measured by Garner et al [37]. For the current frequency range of interest, the real part of *E* is taken to be *E*_*b*1_ = 12.7 *GPa* in the computational simulation. In addition, the complex shear modulus is expressed as

(4.3) $$\mu_{b}=\mu_{b1}+j\omega \mu_{b2}$$

with tan δ_*μ*_ = *μ*_*b*2_ / *μ*_*b*1_. The factor tan *δ*_*μ*_ was determined from experimental measurements [37, 38] and the real part of shear modulus is *μ*_*b*1_ = 3.15 GPa.

In FEA using Comsol, Version 5.3a (Comsol Group, Stockholm, Sweden), the region corresponding to the healthy lung, the collapsed lung and the air were modeled using the *Pressure Acoustics Module*. The soft tissue and the bone were modeled using the *Linear Elastic Material Model (viscoelastic sub-module)*. A *free-free boundary condition* was applied on the torso surface while an *acoustic-structure boundary interaction* was imposed on the lung-muscle tissue interface. An input harmonic excitation of 1 Pa (that corresponds to insonification at the trachea) was applied at the inlet of the trachea.

### 4.2 Simulation of pathological cases

#### 4.2.1 Pneumothorax

##### 4.2.1.1 Three dimensional modeling of lung parenchyma:

**Figure 3.**
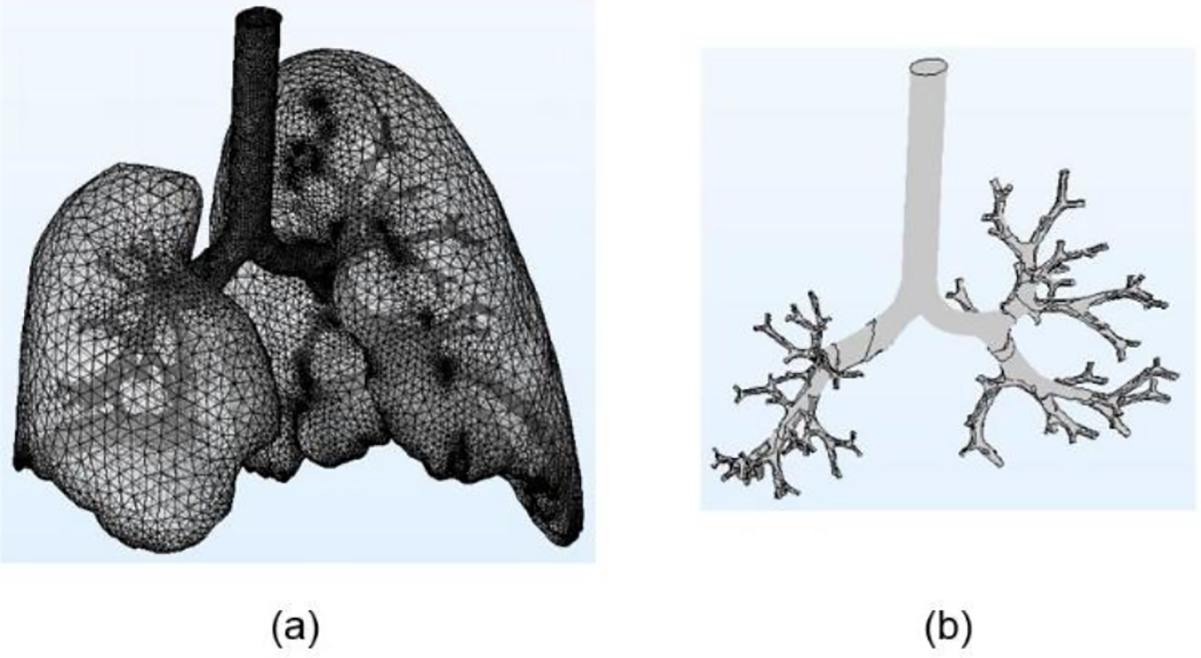
Computational simulation of Pneumothorax: (a) Finite element model of lungs (healthy left lung and right lung affected with Pneumothorax) and (b) 3-dimensional model of airways

To simulate the pathological case of pneumothorax, a 3-dimensional model of the normal right lung was shrunk by 70% in volume (in order to simulate the case of 90% pneumothorax) and fit inside the original lung cavity. The PTX percentage is defined by the ratio of volume of air which occupies the chest cavity outside the lung and the air volume in normal lungs [1, 21]. 3-dimensional models of all the other organs were left unaltered. Figure 3 (a) shows the 3-dimensional model used in the simulation of pneumothorax: the right lung (with an air fraction of *ϕ* = 58.5%) represents the condition of pneumothorax and the left lung (with an air fraction of *ϕ* = 75%) corresponds to a normal lung.

##### 4.2.1.2 Airway acoustics of PTX lung:

According to Peng et al. [9], changes in trans-pulmonary pressure lead to a significant change in the diameter of small airway segments that have a negligible cartilage content. On the other hand, this change in diameter due to the collapse of a lung is negligible for larger airway segments such as the main stem bronchi, trachea and those branches of airways with order number *n* = 31 to *n* = 35. For a small airway segment of order number *n*, the relationship between the change in airway radius *da*^(*n*)^ and the change in trans-pulmonary pressure *dp* is governed by:

…(4.4) $$da^{\left(n\right)}=\frac{\alpha^{\left(n\right)}}{2}dp,\alpha =\frac{A}{A_{0}}$$

In eq. (4.4), *A* is the reduced cross-sectional area due to change in trans-pulmonary pressure and *A*_0_ is the original cross-sectional area.

##### 4.2.1.3 Material properties:

Biot theory [16] was used to compute the material properties of both the healthy lung and the lung affected by pneumothorax. For instance, the density of the normal lung (having volume fraction of air, *ϕ* = 75%) was computed using the Biot theory and was found to be 250 kg/m^3^ while that of the lung affected with pneumothorax (with an air volume fraction of 58.5%) was estimated to be 418.67 kg/m^3^. In addition, the speed of primary waves (*c*_*p*_ or compression wave) and secondary waves (*c*_*s*_ or shear wave) were first estimated at discrete frequencies using Biot theory. Non-linear interpolation (using best curve fit) was then used to compute these properties over a range of frequencies from 100 Hz to 500 Hz.

Impedance of the terminal airways was computed using the Horsfield order number [18], which in turn was computed using the mean diameter. For normal airways the mean diameter of the terminal segments was 2.31 mm corresponding to an order number of 18. In the pneumothorax state, the lung is collapsed and this causes a decrease in the diameter of the airways. Thus the mean diameter for the terminal segments corresponding to the pneumothorax lung was 0.19 mm corresponding to an order number of 8. The impedance of normal lung was computed to be 11.74 – i*(0.55) Pa-s/m while the impedance of the PTX lung was computed to be 17.26 – i*(38.04) Pa-s/m. The terminal impedances thus obtained were then applied to end of the airways as a boundary condition in the computational simulation and the material properties of airways were obtained from Royston et al [25]. Table 1 summarizes the material properties (*c*_*p*_ and *c*_*s*_) that were used in the computational simulation. Note that while the table lists the values of the wave speeds at particular frequencies, in an actual finite element simulation non-linear interpolating polynomials were used to compute the frequency dependent wave speeds.

##### 4.2.1.4 Mode of excitation:

To study the influence of pneumothorax on the sound transmission characteristics of the lungs, excitation in the form of a sinusoidal pressure input at the top of the trachea of amplitude 1 Pa was applied over a range of frequencies from 100 Hz to 500 Hz in the computer simulation to be compared to experimental data obtained as described in Section 3.

#### 4.2.2 Tumor

##### 4.2.2.1 Three dimensional modeling:

**Figure 4.**
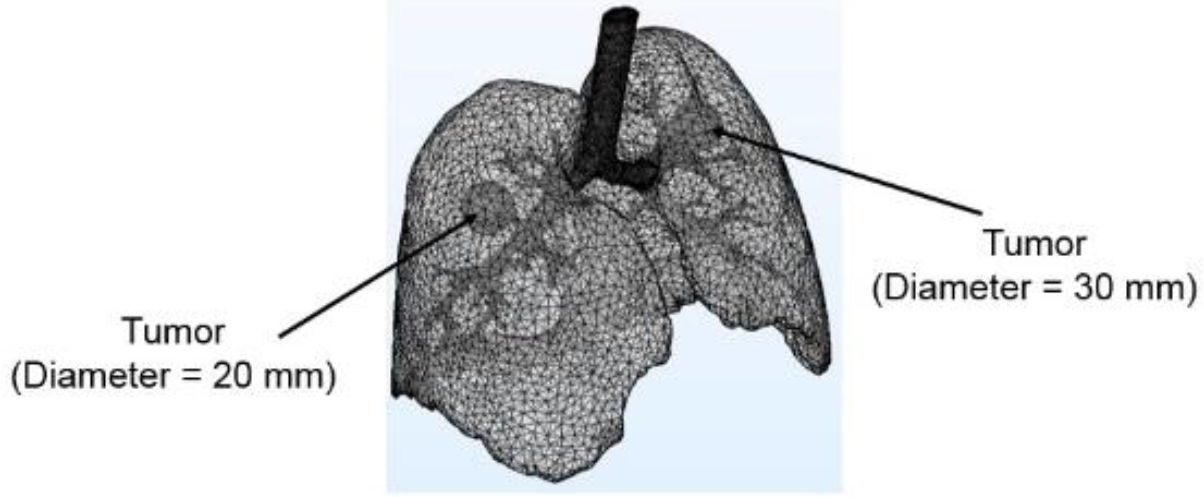
Finite element modelling of tumor

To simulate the pathological case of a tumor, two spherical regions of diameter 3 cm and 2 cm were created as shown in the figure 4 below. As per the guidelines for early diagnosis, detection and staging of lung carcinoma provided by the American Cancer Society [39], a tumor of diameter more than 3 cm but less than 4 cm is considered to be at Stage IB. This category of tumor is considered to be non-malign (the cancer has not spread to nearby lymph nodes or to distant parts of the body).

##### 4.2.2.2 Material properties of the tumor:

The tumors have been modeled as a viscoelastic medium. Current literature does not provide macroscopic shear viscoelastic properties of lung tumor. Based on a clinical study [40] performed on 6 breast carcinoma patients, both the shear viscosity and shear stiffness of the fibro adenomas and carcinomas were larger than those of the normal tissue. In particular, shear stiffness was the parameter that could be used to differentiate a malign tissue from normal tissue. Using that study, the shear stiffness of the tumors was estimated to be 3.33 times the shear stiffness of the normal lung tissue. While the density of the normal lung tissue (with an air volume fraction of *ϕ* = 0.75) was estimated using the Biot theory and was found to be 250 kg/m^3^, the density of tumor was assumed to be that of water (that is, 1000 kg/m^3^). Table 2 lists the compression and shear wave speeds in a tumor [16].

#### 4.3.3 Fibrosis

##### 4.3.3.1 Three dimensional modeling:

The 3-D model used for simulating fibrosis was identical to that of a normal lung, with material properties for the fibrotic lung computed as explained in the following section.

##### 4.3.3.2 Material properties of lung with fibrosis:

Current literature does not have studies pertaining to the macroscopic shear viscosity of lungs affected with fibrosis in the frequency range under consideration in the current study (100 Hz – 800 Hz). In their assessment of six human breast-fibroadenoma patients, Sinkus et al. [40] estimated the shear viscosity and shear stiffness of fibroadenomas and carcinomas at a frequency of 65 Hz and noted that these values were larger than the surrounding normal tissue. In the current study, the shear stiffness increase ratio estimated by Sinkus et al. was extrapolated to the current frequency range of interest. Therefore, the shear stiffness of the fibrotic tissue was assumed to be 1.5 times that of the normal tissue; further, the shear viscosity of the fibrotic lung was chosen such that the shear wave attenuation, governed by the imaginary part of the shear wave number, equals that of the normal lung. In order to simulate the conditions of fibrosis using FEA, the modified material properties in terms of the shear viscoelastic parameters described above were applied to the entire lung parenchyma (as listed in Table 3), while all the other material properties remained the same.

### 4.3.4 Investigation of fibrosis using shear excitation

**Figure 5.**
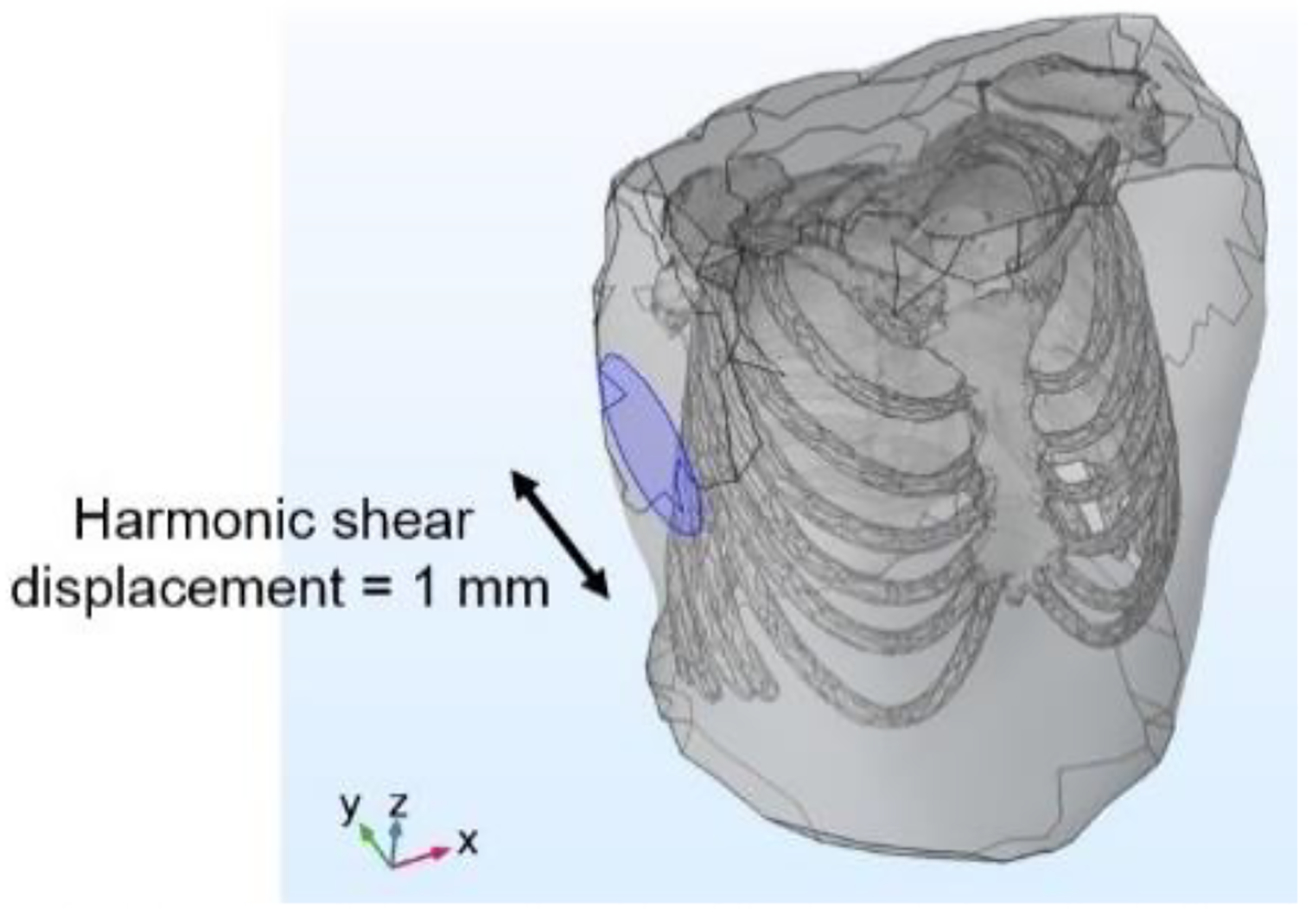
Application of a “shear” excitation on the side of the torso to investigate fibrosis

Insonification is not an effective way to differentiate a healthy lung from a fibrotic lung based on visualization of compression waves [16]. The authors therefore propose the use of shear excitation technique in order to generate and transmit shear waves within the chest so as to effectively differentiate a normal lung from a fibrotic lung. As shown in the fig. 5 below, shear waves were generated by using a “rubbing” motion (or, a sideward to-and-fro excitation) on the chest through the application of a harmonic displacement of 1 mm along the y-direction.

## 5. Results and Discussion

### 5.1 Insonification experimentation on healthy human subjects

**Figure 6.**
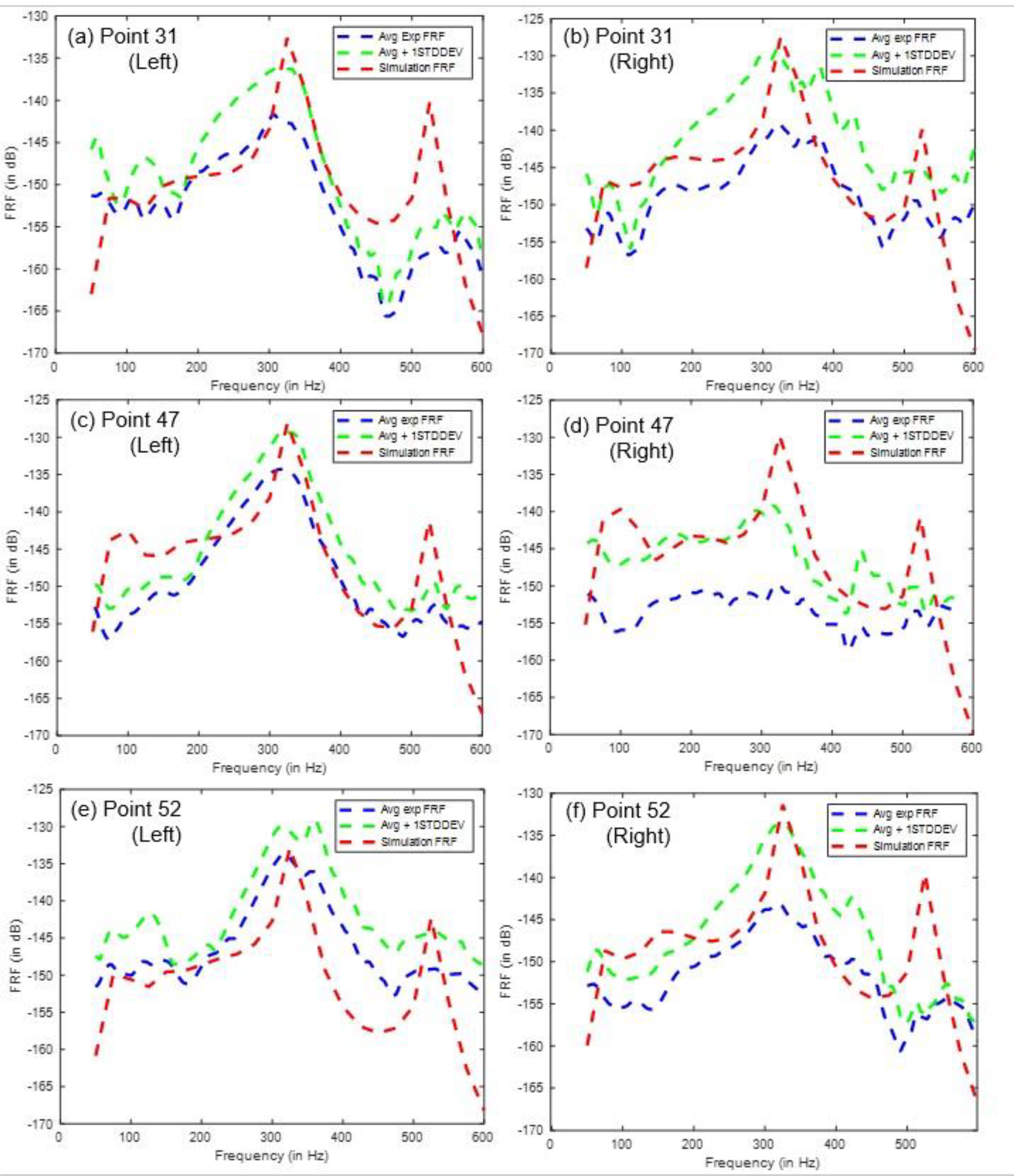
A comparison of results of FRFs for in-vivo experiments involving 3 Human Subjects, with results presented in terms of the Average FRF (blue dashed curve) and Average Plus One Standard Deviation (green dashed curve), both compared against the FRF values obtained from finite element simulation (red dashed curve). **(a,c,e):** comparison of FRF (experiment versus FEA) for points #31, #47 and #52 on the left side of the dorsal side of torso (figure 1(b)); **(b,d,f):** comparison of FRF (experiment versus FEA) for points #31, #47, and #52 on the right side of the dorsal side of torso (Figure 1(b)).

Figure 6 presents a comparison of the average Frequency Response Function (FRF, defined in equation 3.1 as the lung normal surface velocity (in dB) m/s with respect to a 1 Pa input acoustic pressure), shown in blue color, versus average plus one standard deviation values (shown in green) versus the FRF obtained from finite element analysis (represented in red). Averages for experimental FRF were computed by dividing the sum of FRF for each HS at a given experimental frequency by 3. Standard deviation at each frequency was then computed for each of these experimental FRFs using the following mathematical expression:

…(4.4) $$\text{Standard Deviation}=\sqrt{\frac{{\left(\text{HS}1-\text{AVG}\right)}^{2}+{\left(\text{HS}2-\text{AVG}\right)}^{2}+{\left(\text{HS}3-\text{AVG}\right)}^{2}}{3}}$$

From fig. 6, it is observed that the FRF obtained from computational simulation (red curve) follows a similar trend as that of the average plus one standard deviation FRF (green curve) with respect to the spectral magnitudes and features. It is to be noted that this trend is captured more so in the measurements performed on the left side of the torso (fig. 6 (a), (c) and (e)) than in the FRF values computed for points on the right side of the torso (fig. 6 (b), ((d) and ((f)). This is due to the fact that the scanning laser Doppler vibrometer first measured the surface velocity on the left side of the torso posterior before moving on to the points on the right side of the torso posterior, all while the human subject was holding his/her breath. This is believed to have resulted in some deviation in the measured values of surface velocity (in comparison to the computational study, which represents an idealized situation) as some human subjects were unable to hold their breath while the measurements were still being performed on the right side of the torso posterior. This is evident in particular in fig. 6 (d). The observation that the simulation seems to bias to a larger magnitude than the experimental measurements (closer to one standard deviation above it) across many frequencies suggests some small systematic error that is under investigation. Nonetheless, major trends with frequency, such as peaks at ~300 and just over 500 Hz, are generally captured.

In addition, a shift of peaks and valleys of computational results are observed (in comparison to the in vivo human experiments). These shifts could be due to the difference in the size and dimensions of the finite element model as compared to the actual human subjects used in the experimentation. The computational study used a 3-dimensional model that was scaled to the average size of the three human subjects.

**Figure 7.**
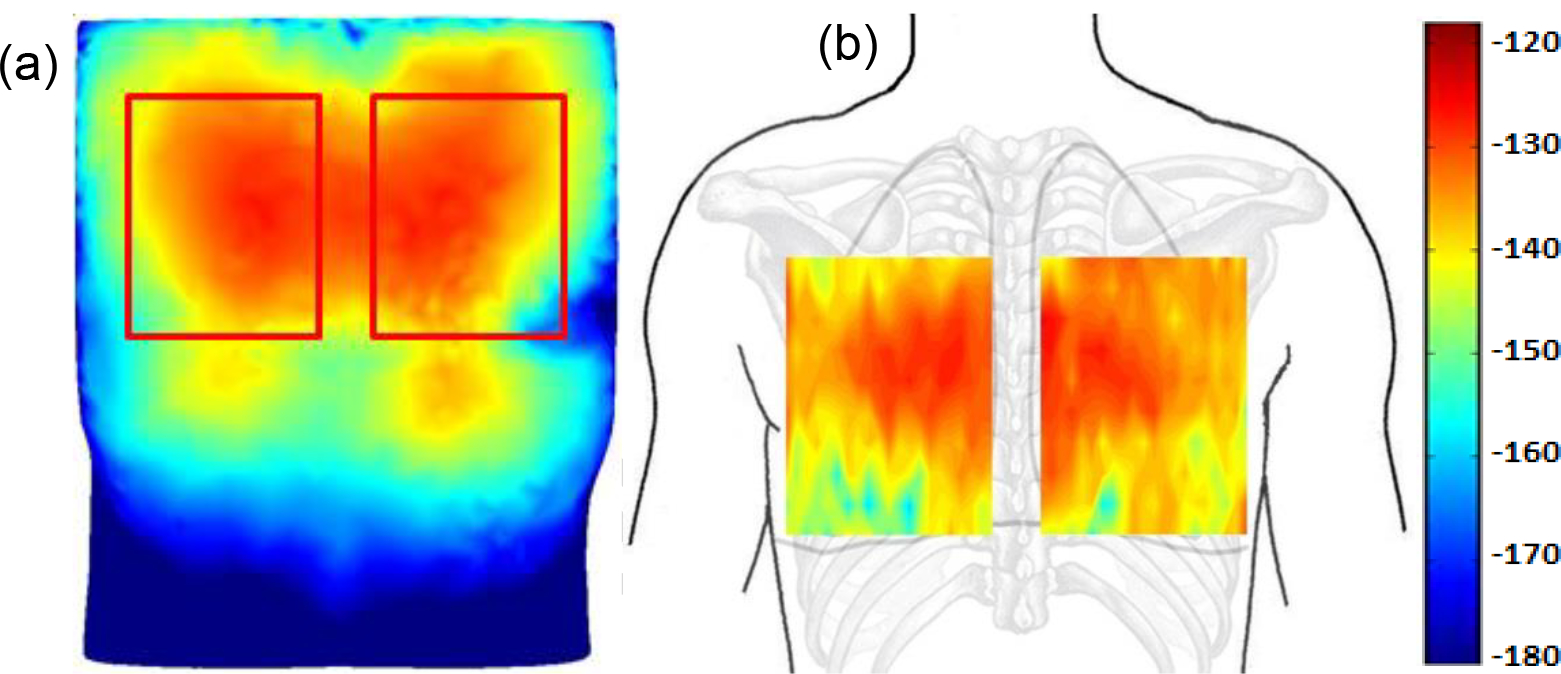
A comparison of the lung normal surface velocity amplitude (in dB m/s per 1 Pa of input acoustic pressure) at 500 Hz for a healthy human torso, computed using (a) simulation and (b) experiment. The rectangle in (a) represents the region of interest for the same areas corresponding to the measurements performed in the experiment as shown in (b).

Figure 7 shows a contour plot of the lung normal surface velocity amplitude (in dB m/s per 1 Pa of input acoustic pressure), at a frequency of 500 Hz for a healthy human subject, computed using (a) FE simulation and (b) in-vivo insonification experiment. The red rectangle (in fig. 7 (a)) represents the Region of Interest (ROI) on the finite element model that corresponds to the same region on the corresponding contour plots shown for the in-vivo experiments in fig. 7 (b). From fig. 7 it is observed that both the experiment and computational simulation show a comparable pattern of velocity amplitude distribution. As expected, the amplitude of velocity is larger over the central areas of the lung (due to the source of excitation being located at the trachea, the path of propagation of excitation is through the airways into the lung parenchyma), the velocity amplitude gradually decays in the peripheral areas. In addition, the pattern of distribution of velocity amplitude observed in the experiment is more mottled than in computational simulation, probably due to the coarser resolution in the experiment and experimental noise that can result in smaller measured velocity amplitudes at some of the scanned points.

The authors note that some models of frequency propagation include a correction for the effect that cheek shunting has on the measurement of the impedance. Alternately, experimental subjects are asked to hold their cheeks in order to minimize cheek shunting. For the current study, however, the authors did not make this correction as the current work made use of measurements of transmission of mechanical wave motion within the lung parenchyma and the torso, rather than making impedance measurements. Corrections to eliminate the effects of cheek shunting could be included in future studies.

### 5.2 Computational simulation of Pneumothorax (PTX)

**Figure 8.**
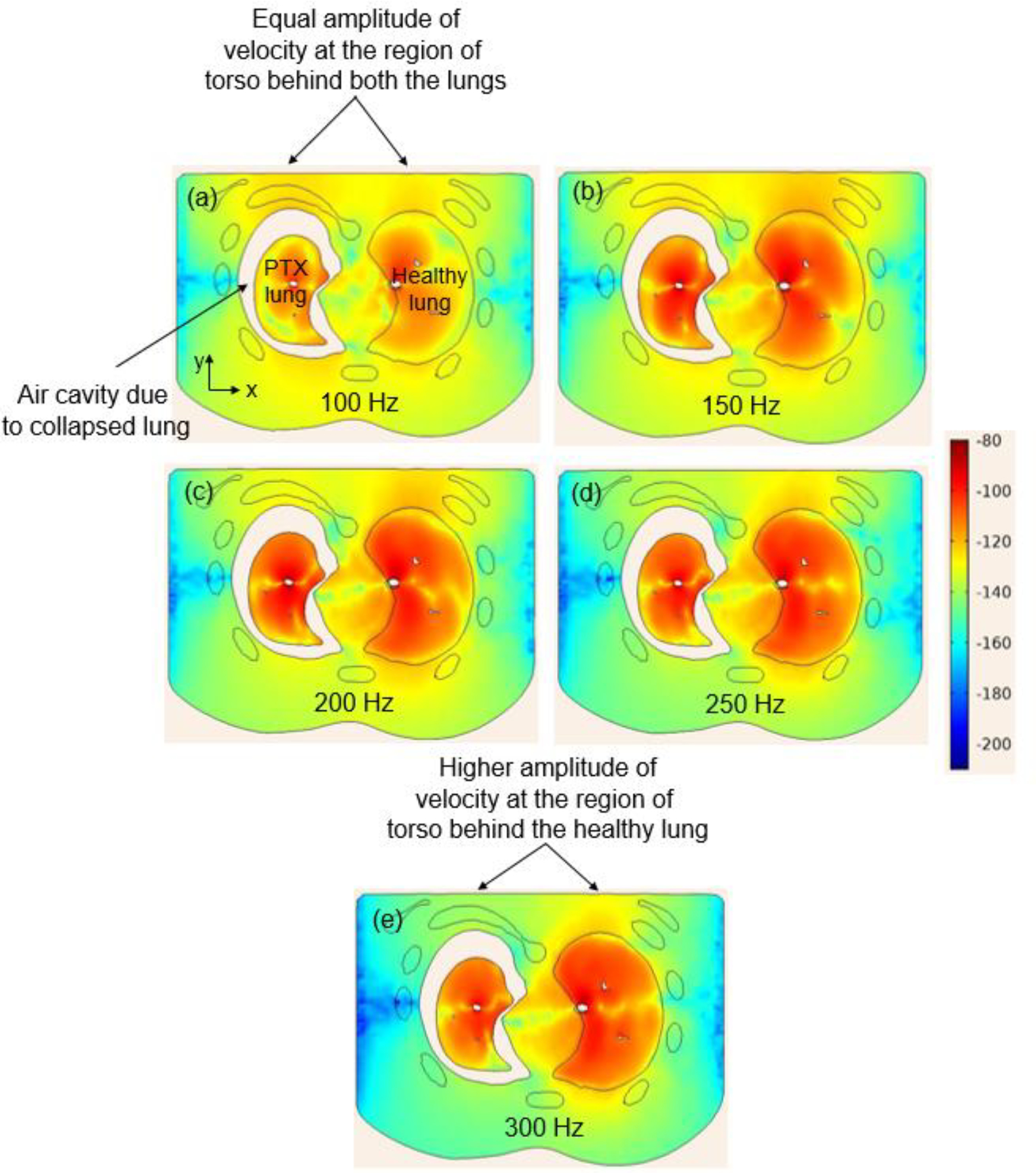
Contour plots of acoustic velocity (in y-direction) shown at a cross section taken in the middle of the torso, as a function of frequency.

The predominant type of waves observed in the lungs due to insonification are compression waves, not shear waves [16]. Figure 8 shows contours of amplitude of y-(i.e., ventrodorsal) velocity (in dB scale) across a cross-section of torso taken at the geometric center (*z* = 0) of the 3D model of the FE study. As the location of measurement of acoustic signal is often at the dorsal side of the torso, an emphasis is therefore laid on the amplitude of wave velocity at the dorsal side of the torso immediately behind the healthy lung and the collapsed lung (due to pneumothorax). It is observed that at lower frequency, the amplitude of velocity is equal behind both the healthy lung and the lung affected with PTX. As the frequency increases, the preferred path of propagation of the compression waves is through the healthy lung more than the lung affected by PTX. This is reflected in the higher amplitude of the velocity at the dorsal side of the torso behind the healthy lung (fig. 8 (e)).

**Figure 9.**
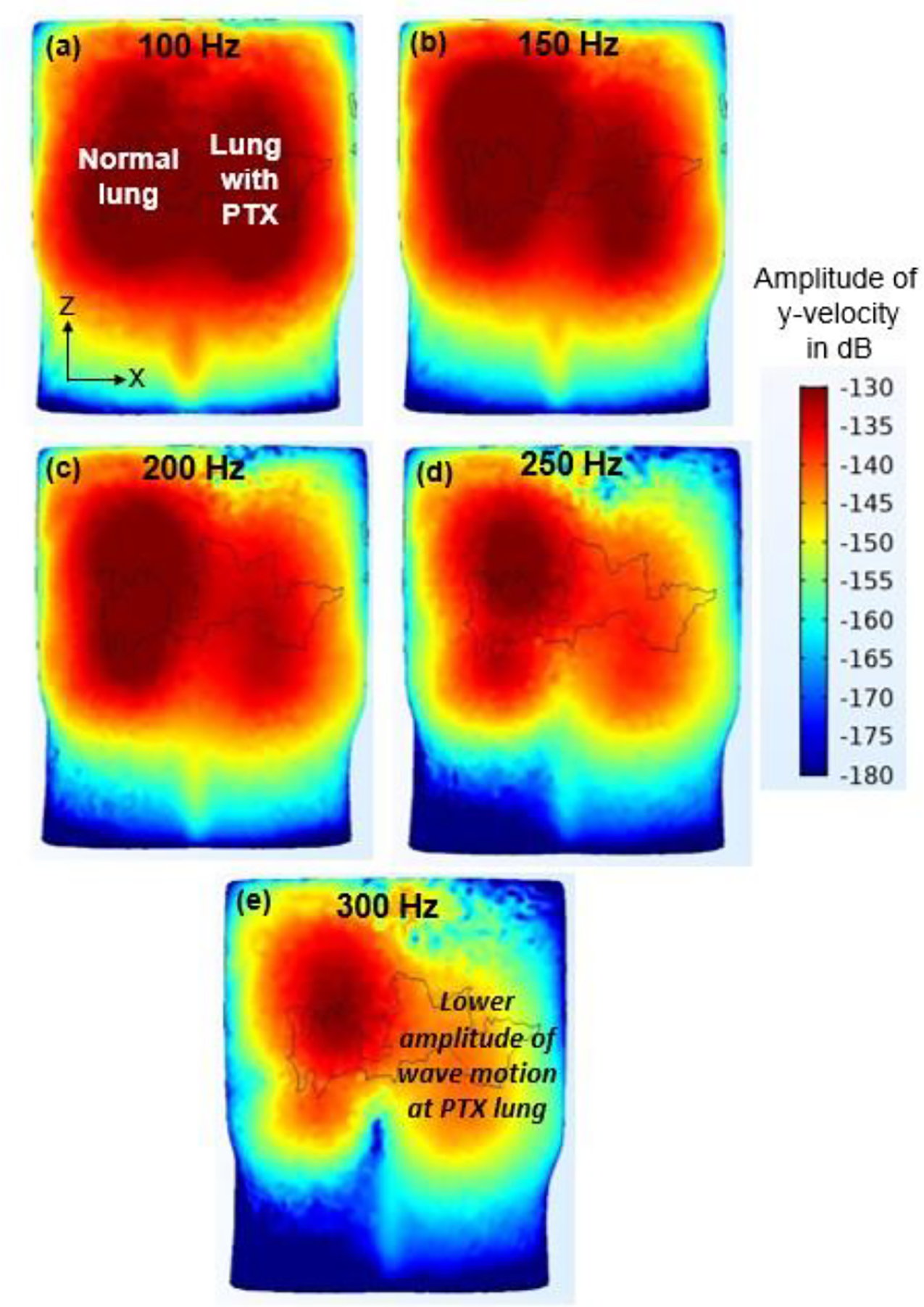
Contour plots of surface velocity in absolute scale (in y-direction) at the dorsal side of the torso as a function of frequency. *Note: y-direction is normal to the zx-plane, pointing outwards from the plane of the image.*

Figure 9 presents another view of the results presented in figure 8, in terms of the surface velocity measured on the torso posterior, on the regions immediately behind the healthy lung as well as the lung affected with PTX. The observations drawn from these contour plots reinforce the same mechanism of wave propagation as a function of increasing frequency, as discussed above where at lower frequencies, the path of propagation of compression waves is through both the healthy lung and collapsed lung. However, as the frequency increases, the path of propagation of the compression waves is predominantly through the healthy lung. These observed spectral trends are also in agreement with human and animal studies of the acoustic effects of PTX [41–43].

Clinically, this finding is significant because it enables non-invasive diagnosis of the presence of PTX by measuring the sound that is transmitted to the chest surface over the lungs. At higher frequencies, the region of the torso surface nearest the lung affected with PTX would give a significantly weaker signal in comparison to a region of torso surface near the healthy lung. A reason for this could be an increased impedance mismatch between the surrounding normal tissue and the collapsed part of the lung (PTX) that has an air cavity. This air cavity imposes a barrier to the propagation of compression waves thereby leading to a weaker signal at the region of the torso behind a PTX lung.

### 5.3 Computational simulation of fibrosis

This section presents the results of computational studies (FE simulations) performed using two techniques, viz., insonification and shear excitation as described in sections 4.3.3 and 4.3.4 respectively.

#### 5.3.1 Insonification

**Figure 10.**
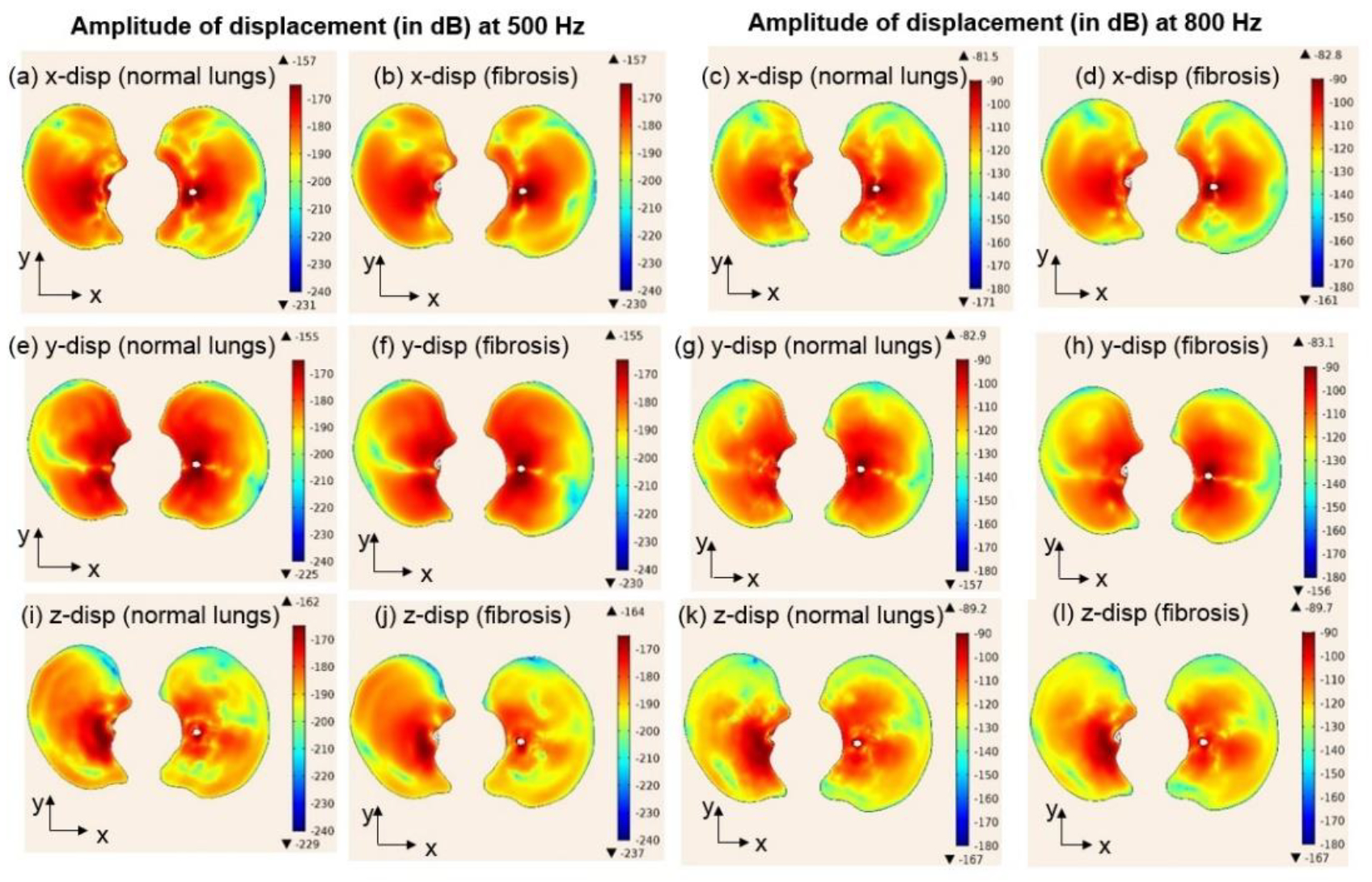
Contour plots of amplitude of displacement (in absolute scale, dB), at two frequencies of 500 Hz ((a), (b), (e), (f), (i), (j)) and 800 Hz ((c), (d), (g), (h), (k), (l)).

Figure 10 presents contour plots of amplitude of displacement (in dB scale) in x-, y- and z-directions at 500 Hz and 800 Hz for both healthy lung as well as the lung affected with fibrosis.

The patterns of propagation of compression waves show insignificant change due to the presence of fibrosis, which confirms the findings of the computational analysis done on porcine subjects [16]. As such, insonification is not an ideal mode of excitation to detect the presence of fibrosis. The authors thus use “shear” excitation (as described in section 4.3.4) to introduce shear waves of lower wavelengths (as compared to the compression waves introduced by insonification).

#### 5.3.2 Investigation of fibrosis using shear excitation

**Figure 11.**
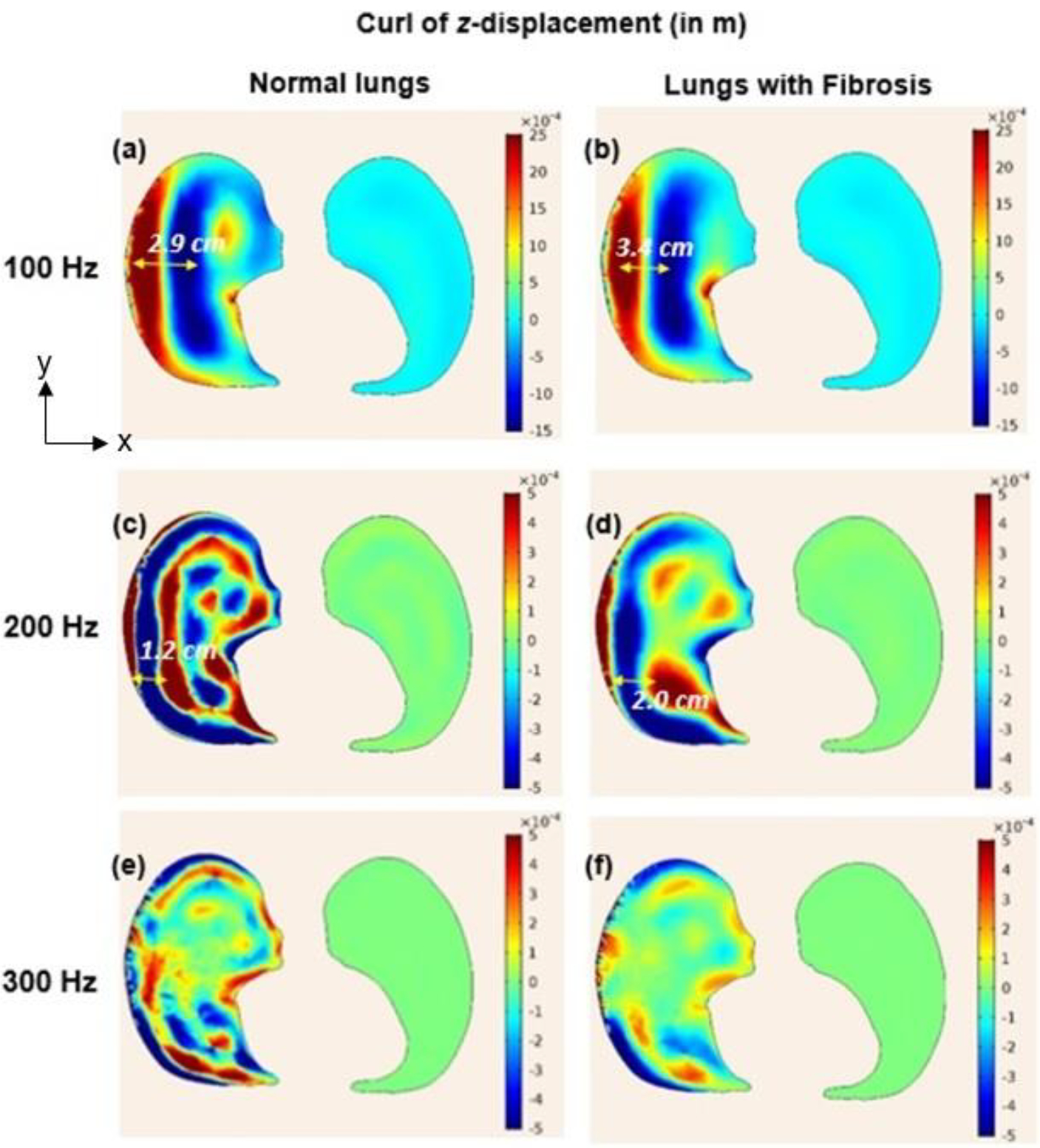
Contour plots of curl of displacement in z-direction (in m) showing propagation of shear waves in normal lung ((a), (c), (e)) and a lung with fibrosis ((b), (d), (f)) at frequencies of 100 Hz, 200 Hz and 300 Hz.

Figure 11 presents the results of “shear” mechanical excitation applied to one side of the torso in order to generate shear waves inside the tissue. The curl operator was applied to eliminate the compression wave component so that the resultant wave form has contributions of pure shear waves. From these contour plots, it is observed that the wavelength of shear waves increases due to fibrosis, due to an increase in the shear stiffness of the lung with fibrosis. Furthermore, this difference is more obvious at lower mechanical frequencies, for example at 100 Hz and at 200 Hz.

From a clinical perspective, this signifies that application of a shear excitation to the torso is more effective method of determining the presence of fibrosis as compared to the use of insonification, which predominantly excites compression waves. For instance, shear excitation can be applied by means of a passive mechanical driver that is placed on the surface of the torso, and the resulting wave motion can be visualized using magnetic resonance elastography technique, as described in [44].

### 5.4 Computational simulation of tumor

**Figure 12.**
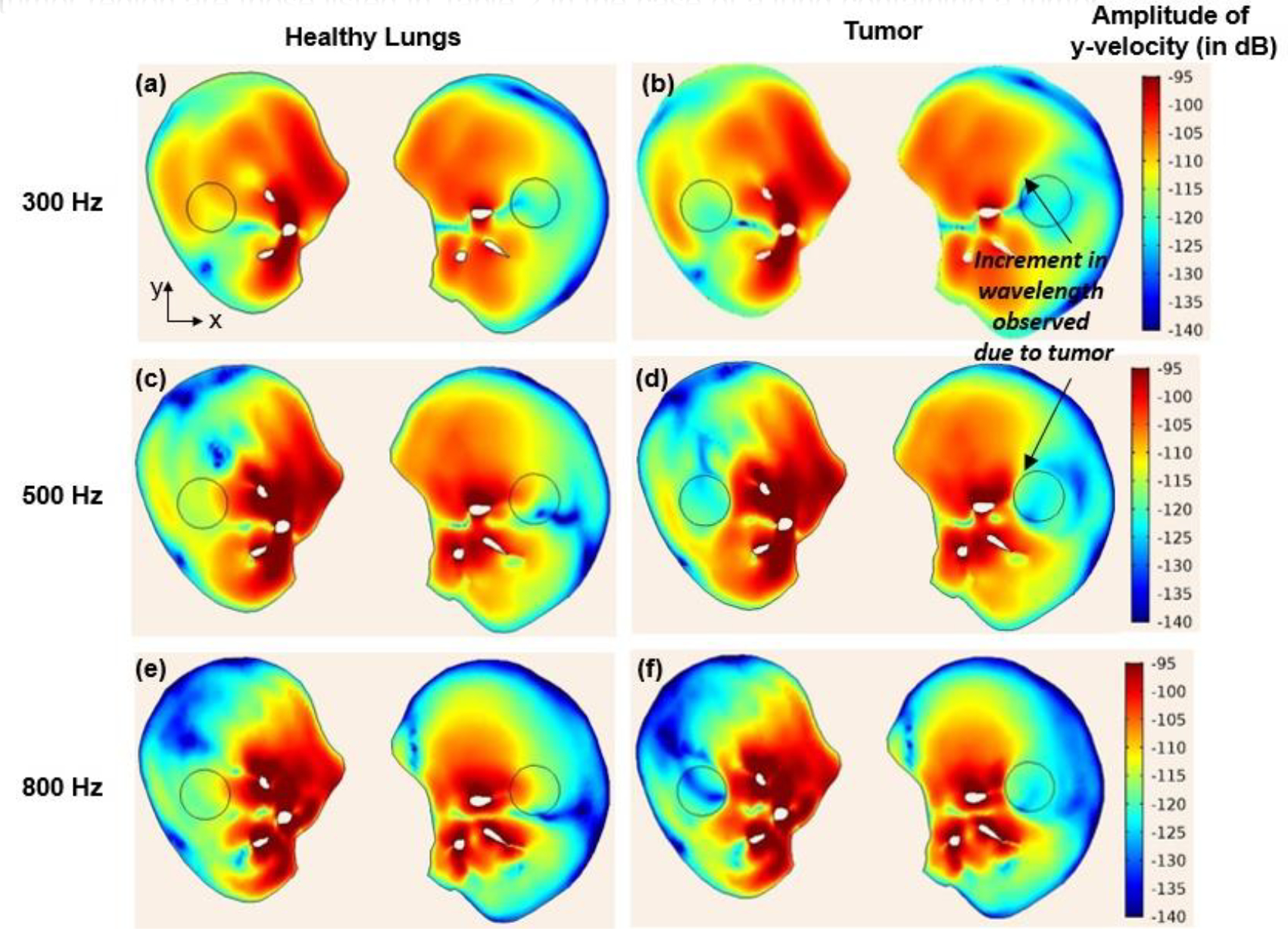
Contour plots of amplitude of velocity (in y-direction), at 300 Hz, 500 Hz and 800 Hz for a healthy lung ((a), (c), (e)) and lung containing tumor ((b), (d), (f)).

Figure 12 presents the results of computational analysis of the pathological case of a tumor, using insonification as a means to introduce compression waves in the lung parenchyma. In these contours, both the healthy (fig. 12 (a), (c), (e)) as well as the lungs with tumor (fig. 12 (b), (d), (f)) show the geometry of a tumor. The mechanical properties of the tumor region are the same as that of a healthy lung parenchyma in healthy case, while the material properties of the tumor region are those listed in Table 2 in the case of a lung containing a tumor.

From the contour plots of velocity amplitude (in dB scale) shown in figure 12, it is evident that the presence of tumor leads to an increase of the wavelength of the propagating compression waves in the vicinity of the tumor region. In addition, due to the impedance mismatch between the tumor and the surrounding lung parenchyma, the velocity amplitudes in the tumor region are diminished, the tumor boundary near the main stem bronchi is more visible possibly facilitating earlier detection of smaller tumors while they are still operable. Several recent and ongoing studies have focused on developing new techniques for enhancing lung MRE [5]. These results suggest that a method that implements airway compression wave acoustic forcing and measures parenchymal displacement or velocity (e.g. using MRE) may be useful for radiation free detection of pulmonary neoplasms.

### 5.5 Limitations of the current study

#### 5.5.1. Limitation 1

**Figure 13.**
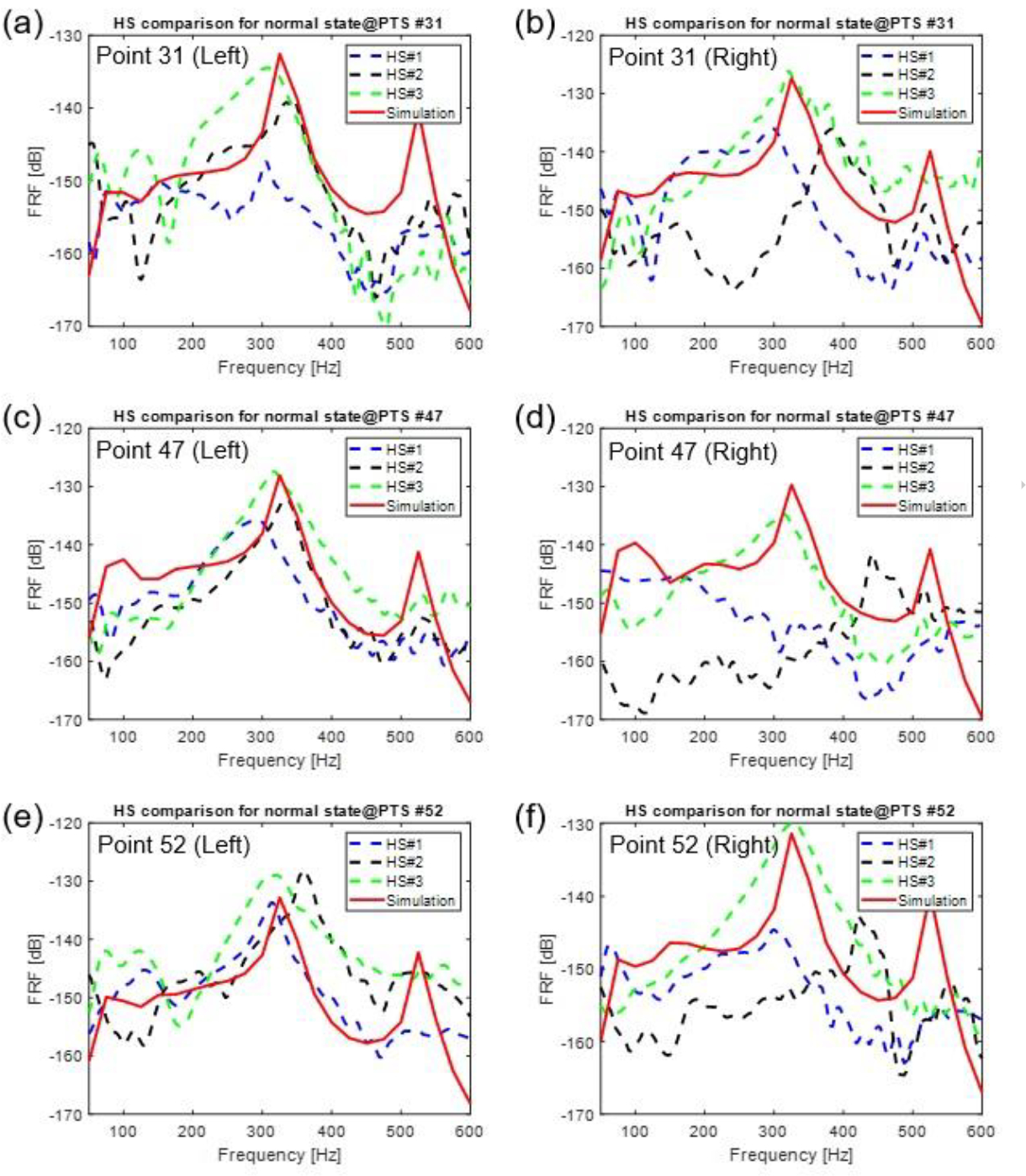
Plot showing the variation of frequency response function (FRF) with mechanical frequency for the three healthy human subjects (dashed curves) versus computational simulation (red curve).

Figures 6 and 13 (Appendix) present a comparison of the Frequency Response Function (FRF) variation with mechanical frequency. While Fig. 6 presents the variation of the FRF in the form of Average and Average + 1 Standard Deviation for the experimental study, Fig. 13 presents variations for all the 3 healthy human subjects (dashed curves) as compared to the computational study. From Fig. 13, it is observed that the experimental variations are closer to the computational (ideal) curves for the measurements made on the left side of the torso posterior, while the measurements made on the right side of the torso posterior show deviation from the computational curve. This is due to the fact that the human subjects were asked to hold their breath still while the measurements were being made by the SLDV (fig. 1), first on the left side, followed by the right side of the torso posterior. HS #1 and #2 were unable to hold their breath while measurements were being made on the right side of the torso posterior, thereby leading to measurements that had contributions made by an unsteady posture and movements made by the human subjects. In the future, due to the advancements made in SLDV acquisition technology, it would be possible to simultaneously measure mechanical motion at all the 48 points on both left and right sides of the torso posterior, therefore leading to a better match between the experimental measurements and computational simulation.

#### 5.5.2 Limitation 2

Prior to experimentation, the three healthy human subjects were not subjected to standard clinical pulmonary tests such as functional residual capacity (FRC) and forced expiratory volume (FEV1). The healthy human subjects were not known to have a medical history of pulmonary diseases and/or respiratory limitations. These tests should be performed during future studies.

## 6. Conclusion

The current work has developed and experimentally validated a comprehensive computational model of sound transmission though a human torso using insonification as a primary mode of excitation. Finite element analysis has been used to generate and visualize contour plots of mechanical wave propagation inside healthy and diseased human lung parenchymae. Finite element analysis enables a non-invasive, subject-based assessment and a quantitative comparison of wave propagation in healthy versus diseased cases, potentially aiding in non-invasive diagnosis of pulmonary ailments such as pneumothorax, fibrosis and localized tumors.

Thus, the present study has used and validated finite element analysis technique as a potential non-invasive, subject-based diagnosis technique that enables a unique visualization of cross-sectional images of wave propagation in the parenchymae, not possible through conventional experimentation/medical examination. In the future, such “maps” of wave propagation in human parenchyma could be potentially used to better quantify the viscoelastic mechanical properties of the lung (such as the frequency dependent shear modulus), by developing a correlation to stiffness maps generated using elastography techniques.

## Figures and Tables

**Figure 2.**
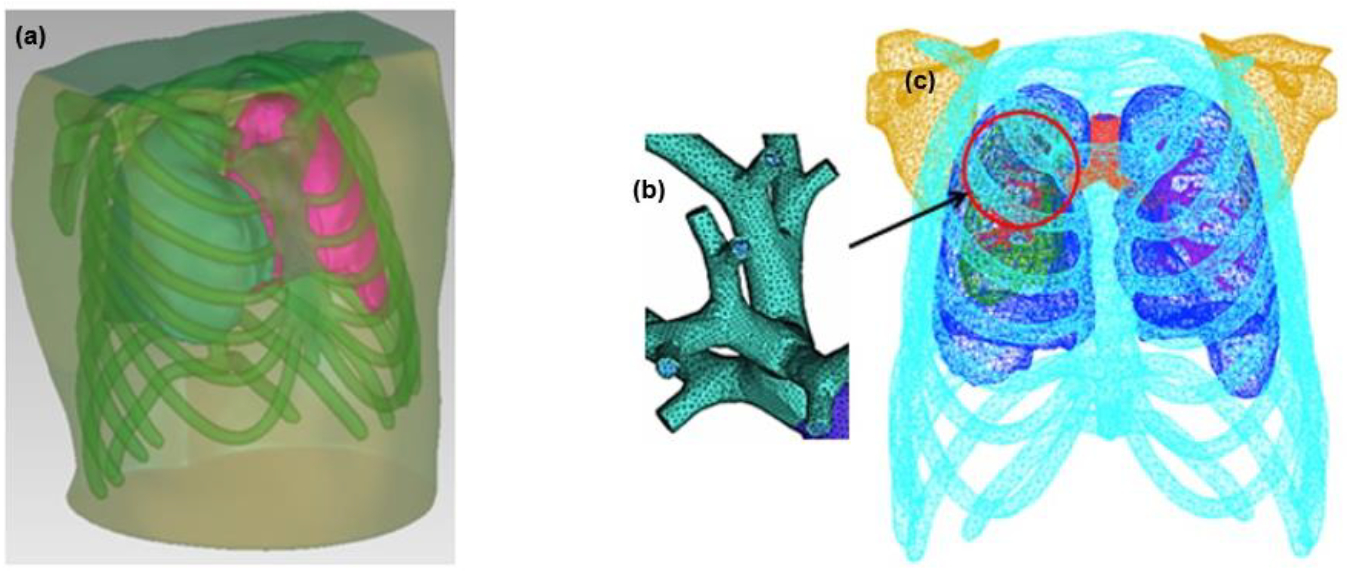
(a) Three dimensional modeling; (b) and (c): generation of finite element mesh

**Table 1.** Sample values of the material properties used in the computational study using finite element analysis

	*Normal Lung*			*PTX Lung*		
Frequency, Hz	(c_p_), m/s	(c_s_), m/s	Impedance (Pa-s/m)	(c_p_), m/s	(c_s_), m/s	Impedance (Pa-s/m)
200	26.02 + i(4.18)	5.62 + i(1.12)	14.14 + i(22.72)	23.47 + i(5.96)	4.36 + i(0.90)	15.16 + i(19.63)
300	27.09 + i(5.64)	5.91 + i(1.27)	15.94 + i(34.32)	25.15 + i(7.90)	4.58 + i(0.98)	16.23 + i(30.22)
400	28.19 + i(6.73)	6.14 + i(1.38)	17.33 + i(40.65)	26.81 + i(9.24)	4.76 + i(1.07)	18.19 + i(45.71)
500	29.24 + i(7.55)	6.33 + i(1.47)	18.76 + i(51.1)	28.32+ i(10.18)	4.91 + i(1.14)	21.04 + i(56.93)

**Table 2.** Compression and shear wave speeds used in the computational study of a lung with tumor

Frequency (Hz)	c_p_ (m/s)	c_s_ (m/s)
300	27.66 + 5.26i	8.26 + 0.91i
500	32.13 + 9.23i	13.91 + 8.09i
800	34.47 + 10.40i	14.78 + 8.54i

**Table 3.** Compression and shear wave speeds used in the computational study of a normal lung and a lung with fibrosis

Frequency, Hz	Normal Lung		Lung with fibrosis	
	Compression wave speed (m/s)	Shear wave speed (m/s)	Compression wave speed (m/s)	Shear wave speed (m/s)
500	29.24 + 7.55i	6.33 + 1.47i	29.66 + 7.56i	7.95 + 2.36i
800	31.81 + 9.12i	6.82 + 1.68i	32.18 + 9.11i	8.53 + 2.69i
